# Association of Medication-Assisted Therapy and Risk of Drug Overdose-Related Hospitalization or Emergency Room Visits in Patients With Opioid Use Disorder

**DOI:** 10.7759/cureus.44167

**Published:** 2023-08-26

**Authors:** Korosh Bahrami, Yong-Fang Kuo, Biai Digbeu, Mukaila A Raji

**Affiliations:** 1 Department of Internal Medicine, University of Texas Medical Branch at Galveston, Galveston, USA; 2 Division of Geriatrics & Palliative Medicine, Department of Internal Medicine, University of Texas Medical Branch at Galveston, Galveston, USA; 3 Department of Preventive Medicine and Population Health, University of Texas Medical Branch at Galveston, Galveston, USA; 4 Department of Biostatistics & Data Science, University of Texas Medical Branch at Galveston, Galveston, USA

**Keywords:** overdose, opioid use disorder, naltrexone, methadone, medication-assisted therapy, buprenorphine

## Abstract

Objective

To examine the differential impacts of medication-assisted therapy (MAT) medications (naltrexone, methadone, and buprenorphine) on drug overdose-related hospitalizations or emergency room (ER) visits in patients with opioid use disorder (OUD).

Patients and methods

A retrospective cohort study was performed on patients 18 years or older diagnosed with OUD, using Optum’s de-identified Clinformatics® Data Mart database. To ensure a new diagnosis of OUD from 2018 to 2019, each patient required one year of continuous enrollment before OUD diagnosis. The primary outcome was the incidence of drug overdose-related hospitalization or ER visits in the follow-up period. Patients were censored at loss of coverage or end of the study (9/30/2020). A multivariable Cox proportional hazard model was built to compare the outcomes across three MAT medications (buprenorphine, methadone, and naltrexone).

Results

Only 10.38% of the 145,317 OUD patients received MAT prescriptions in the 12 months after diagnosis. The majority of MAT users (77.8%) received buprenorphine. At one year of follow-up, the incidence of drug overdose-related hospitalization or ER visits varied by MAT drug type: naltrexone (14.26%), methadone (12.26%), and buprenorphine (10.23%). Compared to methadone drug users, buprenorphine users had a lower risk of negative outcomes (adjusted hazard ratio: 0.84; 95% confidence interval: 0.73-0.97).

Conclusion

Buprenorphine was associated with the lowest risk of drug overdose-related hospitalization or ER visits among the MAT drugs. However, only 10.38% of OUD patients received MAT. Increasing MAT availability to patients with OUD is a key step toward preventing relapse and reducing adverse health outcomes.

## Introduction

Opioid use disorder (OUD) is a growing public health problem affecting over 2.1 million people in the United States alone. The total overdose deaths attributed to opioid involvement rose from 46,802 in 2018 to 68,630 in 2020, while those attributed specifically to prescription opioids increased from 14,139 in 2019 to 16,416 in 2020 [1]. Synthetic opioids other than methadone, mainly fentanyl, have primarily driven drug-involved overdose deaths from 2015 to 2020, which increased six-fold over this period [1]. Deaths from opioid overdose have been particularly exacerbated by the COVID-19 pandemic and the associated public health mitigation measures [1-5].

Three medication-assisted therapy (MAT) medications (methadone, naltrexone, and buprenorphine) have proven effective in treating OUD, reducing mortality in those with OUD, and preventing relapse after recovery [6]. Methadone is a full µ-opioid receptor agonist; naltrexone is a µ-opioid receptor antagonist; and buprenorphine is a partial µ-opioid receptor agonist. The longer overall duration of MAT use is associated with a decreased risk of overdose [7]. In a study of 17,568 opioid overdose survivors, Larochelle et al. found that the use of methadone and buprenorphine reduced opioid-related deaths by 59% and 38%, respectively, compared to non-use; no such association was found with naltrexone, a MAT medication with the shortest use duration [8].

However, general access to MAT for OUD patients is limited, particularly in rural areas of the country. Fully 29.8% of rural Americans live in a county without a buprenorphine provider [9]. Only 11% of patients with diagnosed OUD receive a prescription for MAT [10]. Compounding this issue are ethnic and racial disparities in MAT access. White patients were four times more likely than Blacks to receive a prescription for buprenorphine during an outpatient visit [11]. In addition, Blacks and Hispanics were less likely than Whites to complete treatment [12].

Buprenorphine, methadone, and naltrexone are currently the only FDA-approved medications to treat opioid dependence [13]. While much is known about MAT use and overdose deaths, there is a gap in knowledge about the relationship between the three FDA-approved MAT medications and the risk of drug overdose-related hospitalization or emergency room (ER) visits in patients with OUD. To date, no studies have examined the differential effects of the three MAT medications in reducing drug overdose-related hospitalizations or ER visits in OUD patients. To address this deficiency, we used administrative health claims data from commercial insurance and Medicare Advantage plans to investigate the rates of drug overdose-related hospitalizations and ER visits in OUD patients, according to the type of MAT medication used.

This article was previously presented as an abstract at the University of Texas Medical Branch Forum on Aging on October 25, 2022.

## Materials and methods

Data source

A retrospective cohort study was conducted on patients 18 years or older diagnosed with OUD from 2018 to 2019. The data for this study were obtained from administrative health claims in Optum’s de-identified Clinformatics® Data Mart database, which is one of the largest commercial insurance databases in the United States and which contains patient demographic and clinical information, including information on prescription drugs dispensed and outpatient and inpatient claims. This study was reviewed and approved by the University of Texas Medical Branch Institutional Review Board (approval number: 16-0247).

Study cohort

**Figure 1:**
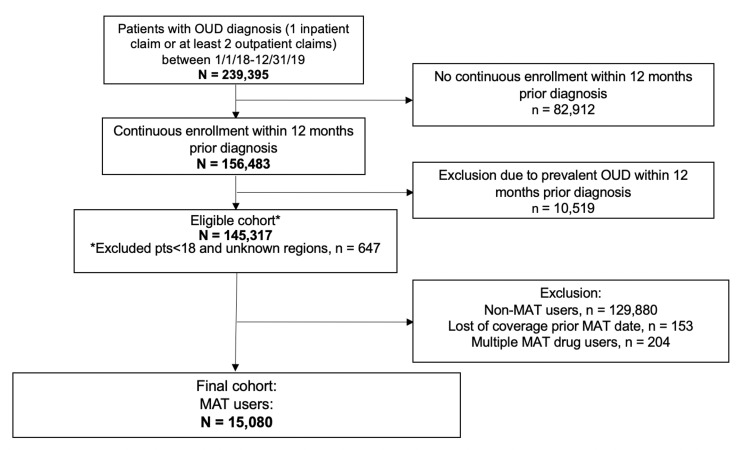
Flowchart for the selection of OUD patients receiving MAT OUD, opioid use disorder; MAT, medication-assisted therapy.

This analysis included patients aged 18 years and older who were diagnosed with OUD from 2018 to 2019. These individuals were identified using the International Classification of Diseases, 10th Revision, Clinical Modification (ICD-10-CM) based on data from the Centers for Medicare and Medicaid Services Chronic Condition Data Warehouse (Table 1). The index date was defined by the first OUD diagnosis from 2018 to 2019. We determined whether each patient received MAT in the 12 months after the index date by looking for national drug codes in pharmacy claims and common procedural terminology codes in medical claims for methadone, naltrexone, and buprenorphine (Table 2). Patients were excluded if they (1) were not continuously enrolled in the 12 months prior to the index date, (2) had an OUD diagnosis in the 12 months prior to the index date, (3) had no MAT prescription after the index date, (4) had lost coverage prior to the MAT prescription, or (5) were prescribed multiple MAT drugs. The final sample size for the analysis was 15,080. Figure 1 shows the process for selecting the cohort for the study.

**Table 1:** International Classification of Diseases, 10th Revision, Clinical Modification (ICD-10-CM) codes based on the CMS Chronic Condition Data Warehouse for OUD and overdose CMS, Centers for Medicare and Medicaid Services; OUD, opioid use disorder.

Disease	
	ICD-10-CM diagnosis
OUD	F1110, F11120, F11121, F11122, F11129, F1114, F11150, F11151, F11159, F11181, F11182, F11188, F1119, F1120, F11220, F11221, F11222, F11229, F1123, F1124, F11250, F11251, F11259, F11281, F11282, F11288, F1129, F1190, F11920, F11921, F11922, F11929, F1193, F1194, F11950, F11951, F11959, F11981, F11982, F11988, F1199, T400X1A, T400X2A, T400X3A, T400X4A, T401X1A, T401X2A, T401X3A, T401X4A, T402X1A, T402X2A, T402X3A, T402X4A, T403X1A, T403X2A, T403X3A, T403X4A, T403X5A, T404X1A, T404X2A, T404X3A, T404X4A, T40411A, T40412A, T40413A, T40414A, T40415A, T40421A, T40422A, T40423A, T40424A, T40425A, T40491A, T40492A, T40493A, T40494A, T40495A, T40601A, T40602A, T40603A, T40604A, T40691A, T40692A, T40693A, T40694A
Drug overdose	T36, T37, T38, T39, T40, T41, T42, T43, T44, T45, T46, T47, T48, T49, T50

**Table 2:** CPT/HCPCS codes for MAT use prescription (methadone, buprenorphine, or naltrexone) and psychotherapy CPT, common procedural terminology; HCPS, Healthcare Common Procedure Coding System; NDC, national drug codes; MAT, medication-assisted therapy.

	CPT/HCPCS codes	NDC codes
Procedure		
MAT	G2067, G2068, G2069, G2070, G2071, G2072, G2073	2810010070
	G2078, G2079, H0020, J0571, J0572, J0573, J0574, J0575	2808120005
	J0592, S0109, J1230, J2315	2808080040
Psychotherapy	90832, 90834, 90837, 90845, 90846, 90847, 90849, 90853, 90785, 90863, 90839, 90840	

Study outcome and covariates

We assessed whether MAT users were diagnosed with a drug overdose-related hospitalization or ER visit at any time after the MAT initiation date until the end of study follow-up (EOF) in September 2020 or until they lost coverage. Table 1 shows the ICD-10-CM codes for the study outcome. Age at the index date was obtained from the Clinformatics® Data Mart database. We examined and adjusted for all conditions 12 months prior to the index date, which was included in the Elixhauser Comorbidity Index. Each condition was examined as a separate covariate. We also adjusted for the sex, race (White, Black, Hispanic, Asian, unknown, or other), and geographic region (Midwest, Northeast, South, and West) of each patient. Finally, we adjusted for psychotherapy counseling at any time after the MAT initiation date until EOF or loss of coverage using common procedural terminology codes in the medical claims (Table 2).

Statistics analysis

Mean (standard deviation) and frequency of patient characteristics and comorbidity were calculated across the three MAT drugs, compared by the ANOVA test for continuous variables and the chi-square test for categorical variables. A Cox proportional hazard (PH) regression model was built to compare the rate of drug overdose-related hospitalizations or ER visits across the three MAT medications. We also adjusted the multivariable model for patient demographics, comorbidity, and a time-dependent variable for psychotherapy. Patients were censored at a loss of coverage or at the end of the study (9/30/2020). The PH assumption for the Cox PH model, visually assessed by a graph of the transformation of the martingale residuals against follow-up time and by the Kolmogorov supremum test, was not violated [14]. All tests of statistical significance were two-sided, with p < 0.05 considered significant, and analyses were performed with SAS 9.4 (SAS Institute Inc., Cary, NC).

## Results

**Figure 2:**
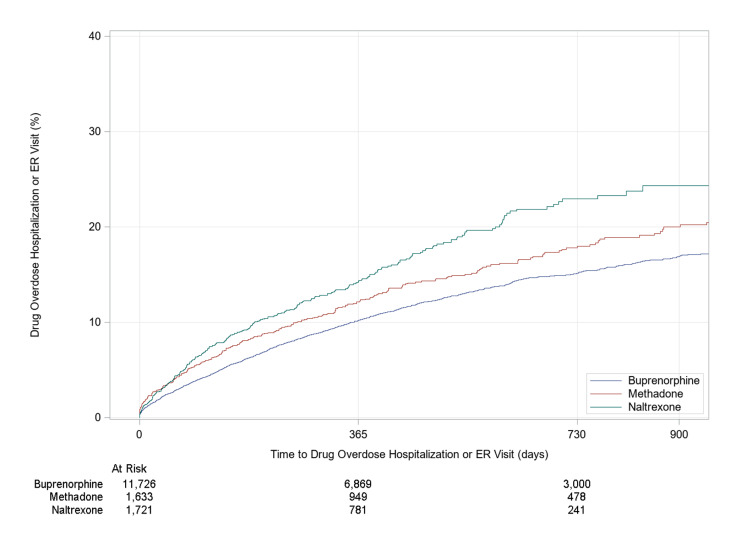
Time to drug overdose-related hospitalization or emergency room (ER) visits by medication-assisted therapy drug used (buprenorphine, methadone, and naltrexone)

Of the 145,317 patients with OUD who met the selection criteria for the study, 10.38% received MAT in the 12 months following their diagnosis (Table 3). The rate of drug overdose-related hospitalizations or ER visits at one-year follow-up was 10.23%, 12.26%, and 14.26% for buprenorphine, methadone, and naltrexone, respectively (Figure 2). At 2.5 years of follow-up, the rate of drug overdose-related hospitalizations or ER visits was 17.09%, 20.49%, and 24.34% for buprenorphine, methadone, and naltrexone, respectively (Figure 2). The different MAT groups varied significantly in total days’ supply and age of patients. Patients in the naltrexone group were given a shorter supply than those in the buprenorphine or methadone group. Patients in the methadone group were older (mean age: 53.50 years) compared to those in the buprenorphine group (mean age: 49.05 years) or the naltrexone group (mean age: 37.93 years). After adjusting for demographic variables and other comorbidities significantly associated with the MAT drug type, the risk of drug overdose-related hospitalization or ER visit among buprenorphine users was 16% lower than for methadone users (adjusted hazard ratio (aHR): 0.84, 95% confidence ratio (CI): 0.73-0.97) (Table 4). Although the risk of drug overdose-related hospitalization or ER visits among naltrexone users was 9% higher than for methadone users (aHR: 1.09; 95% CI: 0.89-1.32), this difference was not statistically significant. Other comorbidities associated with drug overdose-related hospitalization or ER visits included time-dependent psychotherapy (aHR: 1.22; 95% CI: 1.09-1.36), alcohol abuse (aHR: 1.15; 95% CI: 1.02-1.30), cardiac arrhythmia (aHR: 1.45; 95% CI: 1.30-1.63), chronic obstructive pulmonary disease (aHR: 1.33; 95% CI: 1.20-1.47), coagulopathy (aHR: 1.23; 95% CI: 1.03-1.47), depression (aHR: 1.31; 95% CI: 1.18-1.1.45), diabetes (aHR: 1.13; 95% CI: 1.00-1.27), fluid and electrolyte disorders (aHR: 1.41; 95% CI: 1.26-1.59), hypertension (aHR: 1.27; 95% CI: 1.13-1.43), cancer (aHR: 1.75; 95% CI: 1.49-2.07), other neurological disorders (aHR: 1.29; 95% CI: 1.13-1.46), psychoses (aHR: 1.55; 95% CI: 1.32-1.82), valvular disease (aHR: 1.21; 95% CI: 0.03-1.41), and weight loss (aHR: 1.21; 95% CI: 1.03-1.42).

**Table 3:** Descriptive statistics of patient demographics and comorbidities of patients who received buprenorphine, methadone, or naltrexone (n = 15,080) * Total days’ drug supply regardless of continuous enrollment post-OUD diagnosis. ER, emergency room; SD, standard deviation; OUD, opioid use disorder; AIDS, acquired immunodeficiency syndrome; HIV, human immunodeficiency virus.

Characteristics	Buprenorphine, N = 11,726	Methadone, N = 1,633	Naltrexone, N = 1,721	Overall, N = 15,080	p-value
Outcome					
Censored because of end-of-study follow-up	5,645 (48.14)	768 (47.03)	573 (33.29)	6,986	0.0001
Overdose-related hospitalization/ER (event)	1,395 (11.9)	237 (14.51)	259 (15.05)	1,891	
Censored because of loss of coverage	4,686 (39.96)	628 (38.46)	889 (51.66)	6,203	
Age at diagnosis, mean (SD)	49.05 (15.50)	53.50 (15.34)	37.93 (14.69)	48.26 (15.89)	0.0001
Sex					
Female	5,195 (44.3)	703 (43.05)	689 (40.03)	6,587	0.0033
Male	6,531 (55.7)	930 (56.95)	1,032 (59.97)	8,493	
Race					
White	8,525 (72.7)	1,133 (69.38)	1,324 (76.93)	10,982	0.0001
Black	1,128 (9.62)	158 (9.68)	130 (7.55)	1,416	
Hispanic	864 (7.37)	158 (9.68)	133 (7.73)	1,155	
Asian	153 (1.3)	15 (0.92)	27 (1.57)	195	
Unknown or other	1,056 (9.01)	169 (10.35)	107 (6.22)	1,332	
Region					
Midwest	2,233 (19.04)	313 (19.17)	393 (22.84)	2,939	0.0001
North	1,359 (11.59)	217 (13.29)	261 (15.17)	1,837	
South	5,580 (47.59)	694 (42.5)	681 (39.57)	6,955	
West	2,554 (21.78)	409 (25.05)	386 (22.43)	3,349	
Total days’ drug supply*					
1–3 months	4,275 (36.7)	350 (35.86)	1,044 (71.41)	5,669	0.0001
3–6 months	1,859 (15.96)	135 (13.83)	243 (16.62)	2,237	
6+ months	5,513 (47.33)	491 (50.31)	175 (11.97)	6,179	
Post-OUD psychotherapy	3,172 (27.05)	271 (16.6)	912 (52.99)	4,355	0.0001
Elixhauser comorbidity					
Alcohol abuse	1,514 (12.91)	105 (6.43)	914 (53.11)	2,533	0.0001
Cardiac arrhythmia	1,861 (15.87)	308 (18.86)	298 (17.32)	2,467	0.0048
Blood loss anemia	146 (1.25)	40 (2.45)	18 (1.05)	204	0.0002
Congestive heart failure	845 (7.21)	181 (11.08)	57 (3.31)	1,083	0.0001
Chronic obstructive pulmonary disease	3,119 (26.6)	472 (28.9)	312 (18.13)	3,903	0.0001
Coagulopathy	424 (3.62)	118 (7.23)	62 (3.6)	604	0.0001
Deficiency anemia	814 (6.94)	145 (8.88)	86 (5)	1,045	0.0001
Depression	5,477 (46.71)	638 (39.07)	1,068 (62.06)	7,183	0.0001
Diabetes	2,057 (17.54)	374 (22.9)	153 (8.89)	2,584	0.0001
Fluid and electrolyte disorders	1,863 (15.89)	339 (20.76)	300 (17.43)	2,502	0.0001
AIDS or HIV	68 (0.58)	11 (0.67)	11 (0.64)	90	0.8733
Hypertension	5,354 (45.66)	825 (50.52)	505 (29.34)	6,684	0.0001
Hypothyroidism	1,641 (13.99)	264 (16.17)	148 (8.6)	2,053	0.0001
Liver disease	1,227 (10.46)	222 (13.59)	203 (11.8)	1,652	0.0004
Cancer	472 (4.03)	157 (9.61)	34 (1.98)	663	0.0001
Obesity	2,152 (18.35)	324 (19.84)	231 (13.42)	2,707	0.0001
Other neurological disorders	1,208 (10.3)	200 (12.25)	192 (11.16)	1,600	0.0422
Pulmonary circulation disorders	344 (2.93)	77 (4.72)	27 (1.57)	448	0.0001
Peptic ulcer disease, excluding bleeding	263 (2.24)	41 (2.51)	15 (0.87)	319	0.0006
Peripheral vascular disorders	1,124 (9.59)	262 (16.04)	68 (3.95)	1,454	0.0001
Paralysis	142 (1.21)	38 (2.33)	11 (0.64)	191	0.0001
Psychoses	522 (4.45)	36 (2.2)	142 (8.25)	700	0.0001
Renal failure	840 (7.16)	186 (11.39)	45 (2.61)	1,071	0.0001
Rheumatoid arthritis or collagen	1,500 (12.79)	234 (14.33)	99 (5.75)	1,833	0.0001
Valvular disease	685 (5.84)	143 (8.76)	52 (3.02)	880	0.0001
Weight loss	647 (5.52)	139 (8.51)	74 (4.3)	860	0.0001

**Table 4:** Results from the unadjusted and adjusted Cox proportional hazard regression models assessing the effects of MAT drug type on drug overdose-related hospitalization or ER visits MAT, medication-assisted therapy; ER, emergency room; HR, hazard ratio; CI, confidence interval.

Variables	HR	95% CI		p-value
Unadjusted				
Drug (ref = methadone)				
Buprenorphine	0.83	0.72	0.95	0.0071
Naltrexone	1.25	1.04	1.49	0.0146
Adjusted with demographics variables and other covariates significantly associated with the MAT drug category				
Drug (ref = methadone)				
Buprenorphine	0.84	0.73	0.97	0.0161
Naltrexone	1.09	0.89	1.32	0.4169
Age at diagnosis	0.99	0.99	0.99	<0.0001
Sex (ref = male)				
Female	1.12	1.02	1.24	0.0219
Race (ref = White)				
Black	1.15	0.99	1.34	0.0734
Hispanic	1.10	0.93	1.30	0.2931
Asian	1.29	0.90	1.86	0.1680
Unknown	1.12	0.96	1.31	0.1549
Region (ref = South)				
Midwest	1.08	0.95	1.22	0.2455
North	0.88	0.75	1.03	0.1114
West	1.06	0.94	1.19	0.3315
Time-dependent psychotherapy	1.22	1.09	1.36	0.0004
Alcohol abuse	1.15	1.02	1.30	0.0246
Cardiac arrhythmia	1.45	1.30	1.63	<0.0001
Blood loss anemia	0.90	0.66	1.22	0.4798
Congestive heart failure	1.12	0.96	1.31	0.1638
Chronic obstructive pulmonary disease	1.33	1.20	1.47	<0.0001
Coagulopathy	1.23	1.03	1.47	0.0200
Deficiency anemia	0.99	0.85	1.16	0.9077
Depression	1.31	1.18	1.45	<0.0001
Diabetes	1.13	1.00	1.27	0.0464
Fluid and electrolyte disorders	1.41	1.26	1.59	<0.0001
Hypertension	1.27	1.13	1.43	<0.0001
Hypothyroidism	1.06	0.94	1.20	0.3530
Liver disease	1.10	0.96	1.25	0.1660
Cancer	1.75	1.49	2.07	<0.0001
Obesity	0.97	0.86	1.08	0.5505
Other neurological disorders	1.29	1.13	1.46	0.0001
Pulmonary circulation disorders	0.97	0.78	1.20	0.7731
Peptic ulcer disease, excluding bleeding	1.07	0.83	1.38	0.5965
Peripheral vascular disorders	1.13	0.98	1.30	0.0835
Paralysis	1.00	0.73	1.37	0.9953
Psychoses	1.55	1.32	1.82	<0.0001
Renal failure	1.07	0.92	1.25	0.3770
Rheumatoid arthritis or collagen	1.11	0.98	1.26	0.1114
Valvular disease	1.21	1.03	1.41	0.0223
Weight loss	1.21	1.03	1.42	0.0183

## Discussion

The low MAT prescription rate for patients with OUD found here is consistent with findings from previous research. Data from a nationwide study showed that approximately 86.6% of patients with OUD received no prescriptions for any MAT medication [15]. While the rate of MAT prescriptions has steadily increased from 2010 to 2019, in part due to an increase in pharmacy-dispensed buprenorphine, the rate of OUD prescribing remains well below 20% of patients in need [15]. The limited availability of prescribers is a contributing factor to MAT underuse, with 46% of counties having no MAT provider [16]. Improved overall accessibility to MAT for the growing population of patients with OUD requires multilevel approaches. One way to improve access to patient populations in rural areas and underserved inner city sites would be the use of mobile treatment clinics that can be quickly deployed to areas of need, as exemplified by the successful Project Connections at Re-Entry MAT program implemented outside Baltimore City Jail [17]. From a policy level, achieving parity in payments for physical and mental health conditions via the federal Mental Health Parity and Addiction Equity Act is an important step in improving access to overall mental health care and expanding coverage for MAT [18]. Lastly, as in Illinois and other states, expanding Medicaid coverage and eliminating prior authorization for MAT in both Medicaid and private insurance has huge potential to remove barriers to health insurance coverage and expand access to MAT for patients with OUD.

It is not clear why patients who received naltrexone had a significantly shorter days’ supply than those who received either buprenorphine or methadone. One potential explanation is that naltrexone, a µ-opioid receptor antagonist, has the potential to interfere with the treatment of co-occurring chronic pain in patients with OUD, unlike buprenorphine and methadone, which treat both OUD and comorbid pain. Of patients with OUD, 64.4% also have chronic pain conditions [19], making it more challenging to use naltrexone as a long-term maintenance OUD drug compared to the other MAT drugs, which treat both pain and MAT. Our data are consistent with this: 12.79%, 14.33%, and 5.75% of OUD patients with co-occurring rheumatoid arthritis or collagen disease or chronic painful conditions received buprenorphine, methadone, or naltrexone, respectively. Also, OUD patients might be less likely to adhere, at least on a long-term basis, to naltrexone because of its higher cost and the need for full detoxification at drug initiation and titration. Of note, for OUD treatment, the FDA approved only the extended-release long-acting injectable formulation of naltrexone, making it the most expensive of the MAT drugs [20,21]. Thus, buprenorphine and methadone may have longer treatment durations and consequently a greater total days’ supply than naltrexone because they are used as long-term maintenance medications to manage both OUD and the highly prevalent co-occurring pain.

It is unclear why the rate of drug overdose-related hospitalizations or ER visits was lowest in the group receiving buprenorphine. Prior research showed that treatment with buprenorphine improved treatment retention for OUD, so it is possible that better consistency in use among this group could be a contributor [22]. In addition, other studies have reported higher adherence among buprenorphine users, in part due to the lesser severity of opioid withdrawal symptoms when starting buprenorphine [23,24]. Buprenorphine has been shown to be equivalent to methadone in alleviating the severity of opioid withdrawal, but symptoms may resolve more quickly [25]. Moreover, buprenorphine may offer greater flexibility of treatment as well as a lowered risk of adverse effects, such as respiratory depression, because of the ceiling effect [26]. A therapeutic dose of buprenorphine can be achieved in just a few days [27].

## Conclusions

Buprenorphine is associated with the lowest risk of drug overdose-related hospitalization or ER visits among MAT drugs, suggesting a preferential use of buprenorphine (vs. other MAT drugs) as a first-line MAT drug in a shared decision-making process involving patients and clinicians. Regardless, MAT is underused, with only 10.38% of patients with OUD receiving any MAT prescription. Increasing the availability of MAT to patients with OUD is a key step toward preventing relapse and reducing overdose-related ER visits and hospitalizations.
